# Using geographical analysis to identify child health inequality in sub-Saharan Africa

**DOI:** 10.1371/journal.pone.0201870

**Published:** 2018-08-29

**Authors:** Jennifer Yourkavitch, Clara Burgert-Brucker, Shireen Assaf, Stephen Delgado

**Affiliations:** 1 The DHS Program, ICF, Rockville, Maryland, United States of America; 2 ICAP, Mailman School of Public Health, Columbia University, New York, New York, United States of America; Seoul National University College of Medicine, REPUBLIC OF KOREA

## Abstract

One challenge to achieving Millennium Development Goals was inequitable access to quality health services. In order to achieve the Sustainable Development Goals, interventions need to reach underserved populations. Analyzing health indicators in small geographic units aids the identification of hotspots where coverage lags behind neighboring areas. The purpose of these analyses is to identify areas of low coverage or high need in order to inform effective resource allocation to reduce child health inequity between and within countries. Using data from The Demographic and Health Survey Program surveys conducted in 27 selected African countries between 2010 and 2014, we computed estimates for six child health indicators for subnational regions. We calculated Global Moran’s *I* statistics and used Local Indicator of Spatial Association analysis to produce a spatial layer showing spatial associations. We created maps to visualize sub-national autocorrelation and spatial clusters. The Global Moran’s *I* statistic was positive for each indicator (range: 0.41 to 0.68), and statistically significant (*p* <0.05), suggesting spatial autocorrelation across national borders, and highlighting the need to examine health indicators both across countries and within them. Patterns of substantial differences among contiguous subareas were apparent; the average intra-country difference for each indicator exceeded 20 percentage points. Clusters of cross-border associations were also apparent, facilitating the identification of hotspots and informing the allocation of resources to reduce child health inequity between and within countries. This study exposes differences in health indicators in contiguous geographic areas, indicating that specific regional and subnational, in addition to national, strategies to improve health and reduce health inequalities are warranted.

## Introduction

Despite considerable progress, the Millennium Development Goal related to child mortality (MDG 4) was not universally achieved [1]. The under-5 mortality rate in Africa fell from 146 to 65 per 1,000 live births between 1990 and 2012; however, progress has been slow for some countries [2]. One main challenge to achieving the MDGs was the failure to sustain access to quality services for poor communities [3]. Sustainable Development Goals (SDG) include eliminating malnutrition, ending preventable deaths for children under 5, ensuring access to vaccines, and reducing inequality within and among countries by 2030 [4]. In order to achieve those ambitious goals, interventions will need to reach underserved populations. Early in the MDG era, some recognized that it could be possible to achieve the health goals while increasing health inequity, if gains were achieved among the better-off rather than the poor [5]. A subsequent study confirmed the prevalence of pro-rich inequalities in health indicators among 35 countries, while finding that national increases were driven by rapid coverage increases among the poorest people [6]. New tools or methods are needed to accelerate progress; identifying geographic areas that lag behind others could focus attention and resources where they are most needed.

Geographic inequity in health outcomes can occur at national and subnational levels [7]. Given the spatial dimension of health inequity, it is appropriate to analyze health indicators geographically using approaches afforded by geospatial analysis [8], which facilitates the identification of health inequalities, namely, hotspots where coverage of key interventions lags behind neighboring areas, thus aiding precise allocation of resources and interventions where they are most needed [9,10]. Geographic analysis of health indicators can reveal their spatial distribution, which could be influenced by geographic [11] and socio-economic [12] barriers or facilitators, disease patterns [12], and language and culture [13]. By identifying areas of relative high-need, our analysis is a first step to uncovering and addressing causes of health inequity.

### Geographic context of child health inequity for six key indicators

#### Exclusive breastfeeding (EBF)

EBF is recommended for the first six months of life and conveys health benefits to mothers and infants, including reduced morbidity and infant mortality [14,15]. Research on spatial patterns of diarrheal disease in Malawi found that climatic, environmental and geographic effects were mediated by EBF [16]. Interpretation of spatial analysis of EBF draws upon understanding the spatial distribution of natural and cultural features, considering “the context of place” to incorporate social determinants of health [17]. No research has yet examined the spatial distribution of EBF practice across and within a large proportion of countries on the African continent, where EBF prevalence is generally less than 50% [18]. Indicators of health behavior, such as EBF, benefit from geographic analysis that can identify where place-specific facilitators and barriers may exist.

#### Measles and Diphtheria, pertussis, and tetanus (DPT-3) immunizations

Although preventable with immunization, measles is still a leading cause of death for children under 5, and is highly contagious [19]. Diphtheria, pertussis, and tetanus are also vaccine-preventable diseases that contribute to substantial global disease burden among children. In addition to measuring coverage of full protection against these diseases, DPT-3 can be a measure of health system strength because it requires individual follow-up on three occasions.

There is high variability of immunization coverage between and within African countries [20]. Identifying geographic areas of low coverage in order to focus immunization efforts was recommended decades ago [21]. Geographic isolation (remoteness) is a key barrier to equitable vaccine coverage for measles and countries with lower coverage have greater inequity [22]. Vaccination rates correlated with distance to a health center in Niger, where distance was affected by geo-temporal conditions hindering access [11]. That spatial analysis pinpointed optimal locations for new health facilities to improve access for hundreds of thousands of people [11] and exemplifies the utility of subnational spatial analysis of immunization and other child health indicators. No research to date has examined the spatial distribution of immunization coverage across and within a large proportion of African countries.

#### Care seeking

Prompt diagnosis and appropriate management of diarrhea, malaria, and pneumonia is crucial for reducing childhood morbidity and mortality [23] but requires evaluation, i.e., “being seen” by a health provider. Only 28 percent of African children with fever were taken to a public health facility for treatment in 2007 [24]. Only 43 percent of children with symptoms of acute respiratory infection in low-income countries were taken to a health care provider in the last decade [25]. Numerous studies show that distance from the nearest health facility is a significant barrier to seeking care for child illness, both because of the direct cost of travel and the indirect cost of time lost during travel [11,23,26–28], which has led to initiatives to provide good-quality care in communities [29]. There is some evidence that improving accessibility to health care in low- and middle-income countries can improve health equity by reducing socioeconomic gaps in care [30]. No study has yet examined care seeking across much of Africa and within a large proportion of its countries.

#### Stunting

Stunting is a sign of undernutrition and repeated infections that occurred during pregnancy and the first two years of life, and is identified by a height that is below two standard deviations from the median WHO Child Growth Standard [31]. Stunting increases lifetime risks of impaired health and affects educational and economic performance [32], with consequences for human capital and social progress [33,34]. Stunting is also the best proxy measure for child health inequity because it reflects multiple environmental aspects of children’s health and development [35,36], including feeding behaviors and socio-economic factors. Poor living conditions are main determinants of stunting; poverty is more detrimental for height than for weight [34,37]. Research from the Democratic Republic of Congo (DRC) shows that malnutrition is spatially structured—higher in rural areas than in urban centers—and overlaps with political and economic factors: malnutrition is high in provinces where mining is the main industry, where there is conflict, and where food is produced, likely due to the economic benefit of selling food versus consuming it [38].

Data from 1995 to 2003 indicate a stunting prevalence range from 12 to 30 percent among 16 sub-Saharan African countries [36]. An analysis of 47 developing countries concluded that focusing on reducing malnutrition overall would not necessarily redress inequity [39]; that would require targeting efforts to specific groups. To the extent that poverty clusters geographically, for example, in urban slums and remote rural areas, there is a role for geographic analysis to identify where to focus resources. No research has yet examined the spatial distribution of stunting across and within a large proportion of countries on the African continent.

#### Under-5 mortality

From 1990 to 2012, the under-5 mortality rate in sub-Saharan Africa declined nearly 45 percent, from 177 to 98 deaths per 1,000 live births [40]. However, that is still the highest regional mortality rate in the world for children: 3.2 million children under 5 died in sub-Saharan Africa in 2012, accounting for nearly half of global under-5 deaths [39]. Moreover, an increase in under-5 mortality was observed in several African countries during the 1990s; data from five of those countries indicate that mortality increases were concentrated in specific population subgroups, whose education level and urban/rural residence varied by country [41]. A study of Demographic Health Survey (DHS) data from the DRC in 2007 found unexpected geographic patterns in under-5 mortality, and highlights subnational areas where a potential confluence of individual, household and environmental elements affecting child mortality may be spatially clustered [42]. Studies in rural Tanzania and Burkina Faso found that physical access to health facilities was associated with child mortality [43,44]. A study of the spatial distribution of under-5 mortality across and within 20 African countries using data from the late 1970s to the early 1990s ascribed geographic patterns to similar disease environments in eastern Africa and to economic development along the coast of western Africa [12].

### Purpose of analysis

The purpose of this study is to identify geographic inequalities in six child health indicators across 27 countries in sub-Saharan Africa. Targeting health interventions to high-need populations can be a cost-effective approach to reducing child mortality and can reduce inequities in coverage between the most and least deprived geographic areas [45], but this strategy requires first knowing where high-need populations are located by identifying geographic inequalities in health indicators. Geographic analysis can be used to analyze health care access and to direct resources where needed [46]. Monitoring progress across geographic and socioeconomic indicators between and within countries can determine if programs and policies are benefiting the poorest people [47] and are implemnented where most needed, thus contributing to improved and equitable health outcomes. The spatial autocorrelation analyses presented here augment research that identifies and explores spatial implications for child health. We present new visualizations of subnational estimates of six key child health indicators. These analyses facilitate the identification of hotspots of low coverage or high need, and can be used to allocate resources effectively to reduce health inequities between and within countries. Sub-Saharan Africa is an important region to examine because of the low coverage, high need, and uneven progress on addressing coverage of key indicators. Our timeframe, 2010–2014, provides an important check on health inequalities by examining coverage differences among subnational areas during the last third of the MDG monitoring period, and a baseline benchmark for the SDG era.

## Methods

### Data description

We conducted descriptive, geographical, secondary analyses from de-identified, publicly available datasets from The Demographic and Health Survey (DHS) Program and did not require Institutional Review Board (IRB) approval. Each DHS survey obtains IRB approval [48]. Our analyses use data from surveys conducted in 27 selected African countries between 2010 and 2014, with methods similar to those used in our previous work [49]. These countries are: Benin, Burkina Faso, Burundi, Cameroon, Republic of Congo, Côte d’Ivoire, Democratic Republic of Congo (DRC), Ethiopia, Gabon, The Gambia, Ghana, Guinea, Kenya, Liberia, Malawi, Mali, Mozambique, Niger, Nigeria, Rwanda, Senegal, Sierra Leone, Tanzania, Togo, Uganda, Zambia, and Zimbabwe. The units of analysis used for this analysis are DHS regions, which correspond to administrative level-one areas (e.g., provinces) or a combination of such areas within each survey country. The data are statistically representative estimates of each indicator of interest for the population within each DHS region at the time of each survey.

The DHS Program surveys used in this analysis were conducted using standard approaches which are designed to allow for comparison within a country at DHS regional-level and between countries or regions. The survey populations are sampled using a standard two-stage method where the first stage involves the selection of enumeration areas (probability proportional to size) generally drawn from census files. The second sampling stage involves the random selection of individual households within each selected enumeration area. The data are collected and verified using rigorous data quality measures [50].

We used the survey data to compute six key child health indicators: EBF, measles vaccination, DPT3 vaccine coverage, care seeking behavior, stunting prevalence, and under-five mortality rate, defined in Table 1. We constructed the key indicators according to The DHS Program definitions and we calculated indicator estimates for the country as a whole as well as for each DHS sample region. We used sampling weights and a stratified sample design to produce estimates, confidence intervals and standard errors with Stata (v.14. College Station, TX: StataCorp LP).

**Table 1:** Child health indicators in this analysis.

Indicator	Definition
Exclusive breastfeeding (EBF)	Proportion of last-born infants under age 6 months who are living with the mother and breastfeeding and have not had any water, liquids, or solids in the day or night preceding the interview
Measles vaccination	Proportion of live children age 12–23 months who received the measles vaccination at any time prior to the survey
DPT3 vaccine coverage	Proportion of live children age 12–23 months who received three doses of DPT vaccine at any time prior to the survey
Care seeking behavior	Proportion of children age 0–59 months who had cough, diarrhea, or fever in the last two weeks and sought treatment
Stunting prevalence	Proportion of *de facto* children age 0–59 months whose height-for-age z-score is more than 2 standard deviations below the median on the WHO 2006 international reference standard
Under-five child mortality rate*	Number of deaths among children under age 5 in the five-year period preceding the survey per 1,000 live births

*The DHS Program uses a synthetic cohort life table approach to directly estimate the under-five mortality rate [50].

We obtained DHS survey region borders from The DHS Program Spatial Data Repository [51]. Each country had between 3 and 26 regions (Table 2). We merged individual country shapefiles into a single feature class using ArcGIS (v.10.2 Redlands, CA). We cleaned topographic polygon overlap errors with ArcGIS; some small gap slivers remained in the dataset. The final feature class comprised 255 polygons representing every survey region from the 27 surveys. We joined indicator estimates to the polygon dataset and constructed a spatial weights matrix in ArcGIS with the Spatial Statistics Tools using a “Contiguity Edges Corners” (i.e., Queen) conceptualization of spatial relationships and row standardization. The weights matrix defines spatial neighbors as any areas with either a border (edge) or a corner touching.

**Table 2:** Country survey year, and number of DHS survey regions in this analysis.

Country	Survey Year	Number of Survey Regions
Benin	2012	12
Burkina Faso	2010	13
Burundi	2010	5
Cameroon	2011	12
Congo	2012	12
Cote d'Ivoire	2012	11
Democratic Republic of the Congo (DRC)	2014	11
Ethiopia	2011	11
Gabon	2012	10
Gambia	2013	8
Ghana	2008	10
Guinea	2012	8
Kenya	2008–09	8
Liberia	2013	5
Malawi	2010	3
Mali	2013	6
Mozambique	2011	11
Niger	2012	8
Nigeria	2013	6
Rwanda	2010	5
Senegal	2011	14
Sierra Leone	2013	4
Tanzania	2010	26
Togo	2013–14	6
Uganda	2011	10
Zambia	2013	10
Zimbabwe	2011	10

### Data analysis

**Fig 1:**
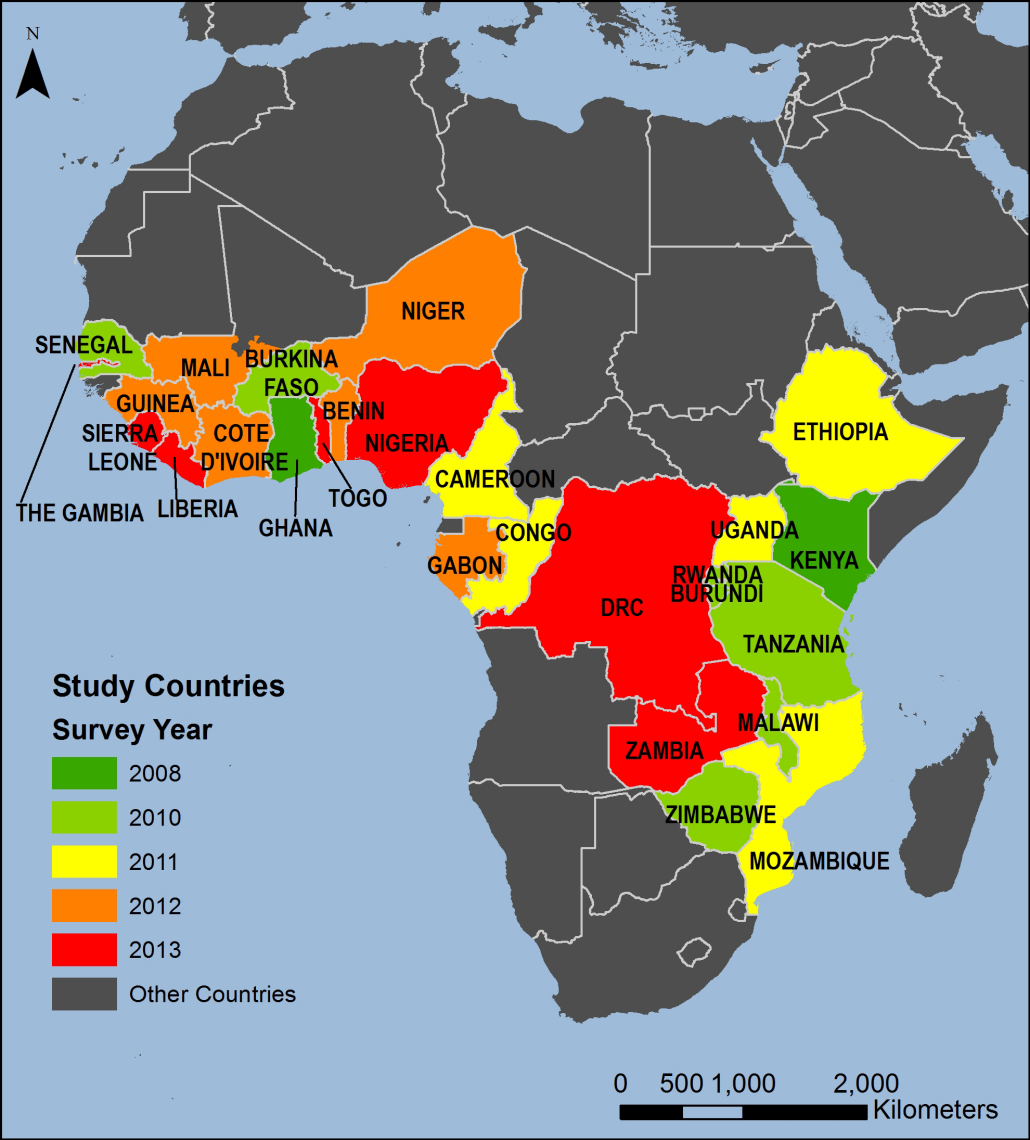
Reference map.

We analyzed indicators in 255 survey regions across 27 countries (Table 2 and Fig 1). Every region had at least one neighbor. Exploratory spatial data analysis (ESDA) methods visualized and measured the spatial autocorrelation between and among regions that are spatially contiguous. We first visualized the spatial patterns of the selected child health indicators across the selected areas to provide a quantitative assessment of each indicator both within and across national boundaries. We then identified areas with statistically significant clustering of high values (hotspots) or low values (coldspots), as well as spatial outliers, for the selected indicators. This analysis provides a statistical intra- and inter-country assessment of relatively high and low performing areas with respect to geographically proximal areas.

### Global and local spatial autocorrelation of child health indicators

We constructed thematic maps of child health indicators in ArcGIS. This analysis allows quantitative assessment of each indicator and its uncertainty estimate both within and across national boundaries. Nearby locations tend to be more alike than locations that are far away from one another [52]. Spatial autocorrelation statistics measure the degree to which a sub-region is similar to or different from its neighboring sub-regions for a particular indicator. Spatial autocorrelation may be evaluated globally as well as locally. Global measures summarize spatial autocorrelation across a study area, while local measures evaluate localized spatial autocorrelation within a study area.

The Moran’s *I* statistic is a measure of global spatial autocorrelation or “clustering” [53], A Moran’s *I* statistic can range from -1 to 1; a value close to zero indicates that there is no or random spatial clustering across the study area as a whole; a positive statistic indicates spatial clustering, where neighboring sub-regions tend to have similar indicator values; and a negative statistic indicates that neighboring sub-regions tend to have different indicator values. The local indicator of spatial association (LISA) for Moran’s *I* is a measure of local spatial autocorrelation, which indicates the “presence or absence of significant spatial clusters or outliers for each location” in a dataset [54].

We conducted the Global Moran’s *I* and LISA analyses with GeoDa software (v.1.6.7) using the Spatial Autocorrelation (Univariate Moran’s *I*) tool and the Local Indicator Spatial Association (LISA) (Univariate Local Moran’s *I*) tool, respectively. The LISA analysis produces a spatial layer that presents up to five types of spatial association and outliers [49]:

- Not significant: Areas with no statistically significant spatial autocorrelation (at *p*≤ 0.05).
- High-high: High values surrounded by other high values. (Note: these values are not necessarily “high” in absolute value but they are the high values from this dataset.)
- Low-low: Low values surrounded by other low values. (Note: these values are not necessarily “low” in absolute value but they are the low values from this dataset.)
- Low-high: Low values surrounded by high values.
- High-low: High values surrounded by low values.

## Results

### Map interpretation

**Fig 2:**
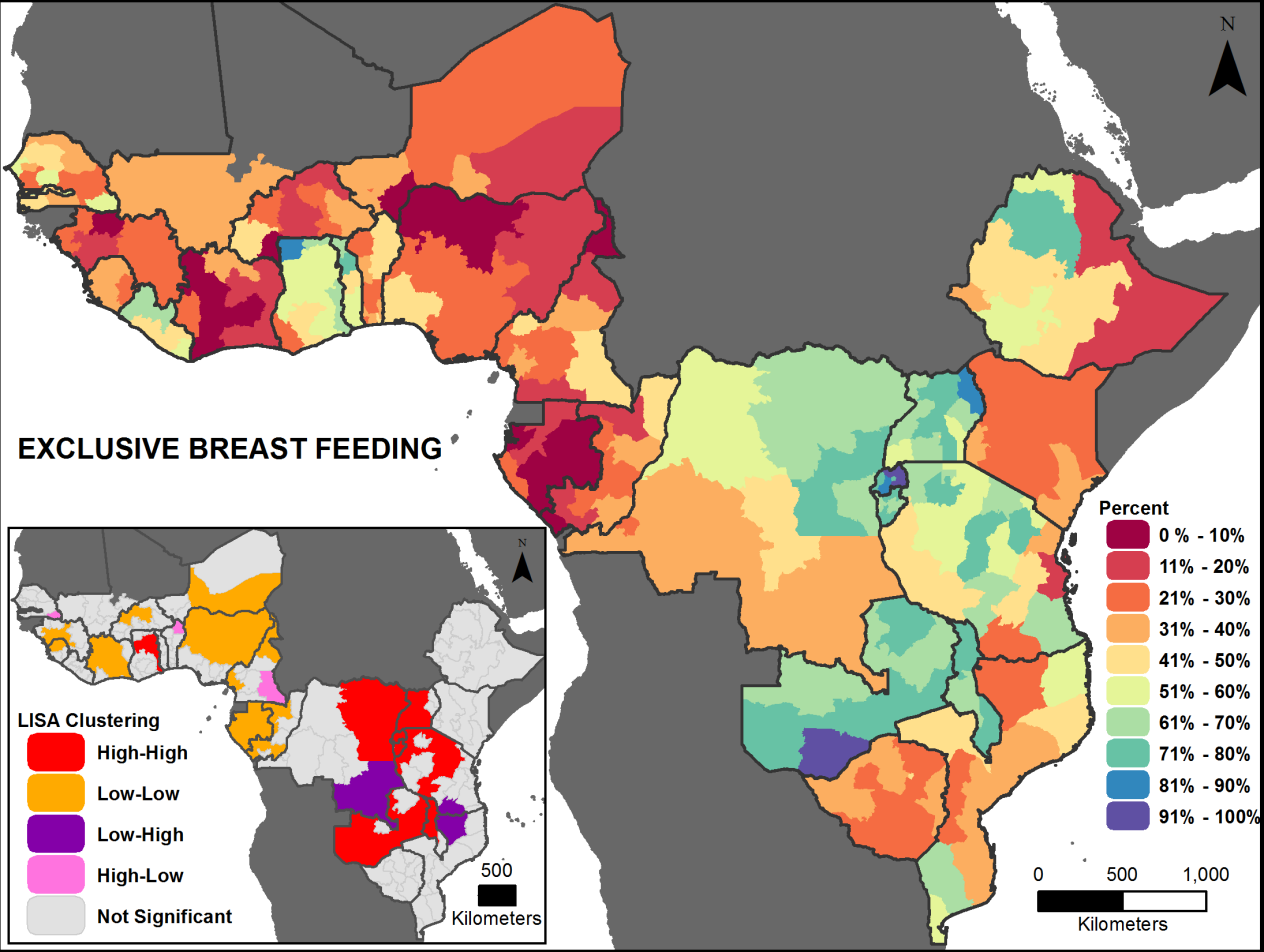
Exclusive breastfeeding prevalence map with LISA analysis.

**Fig 3:**
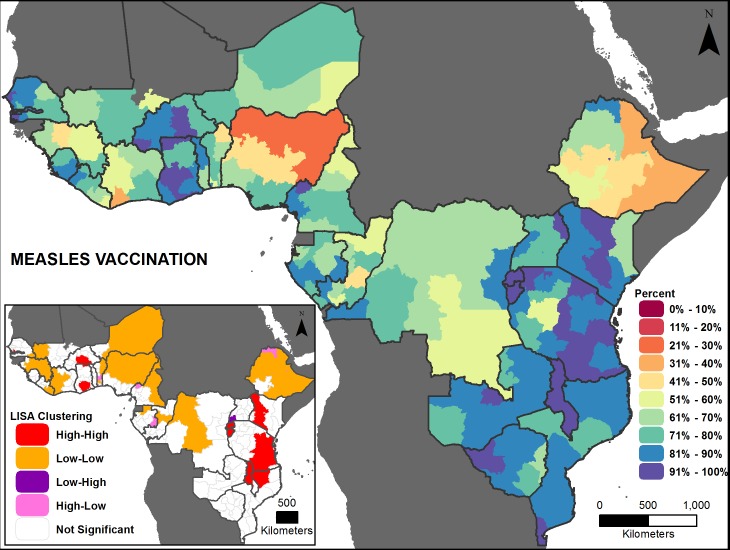
Measles vaccination prevalence map with LISA analysis.

**Fig 4:**
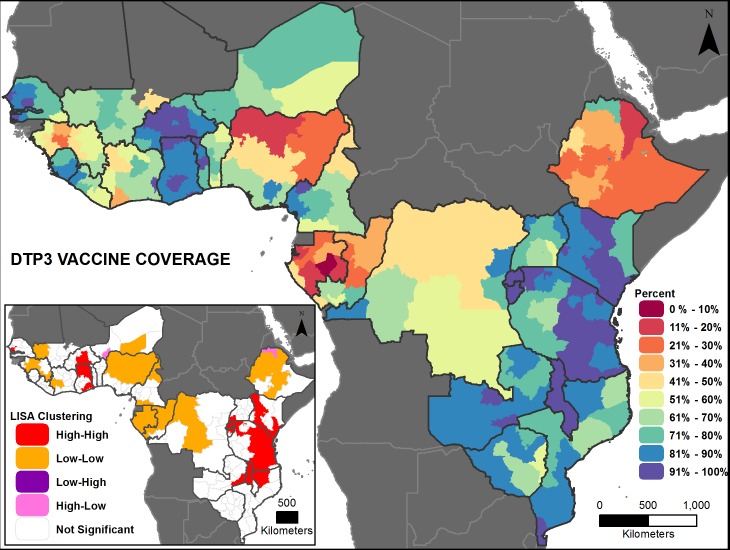
Diphtheria-pertussis-tetanus (DPT3) vaccination prevalence map with LISA analysis.

**Fig 5:**
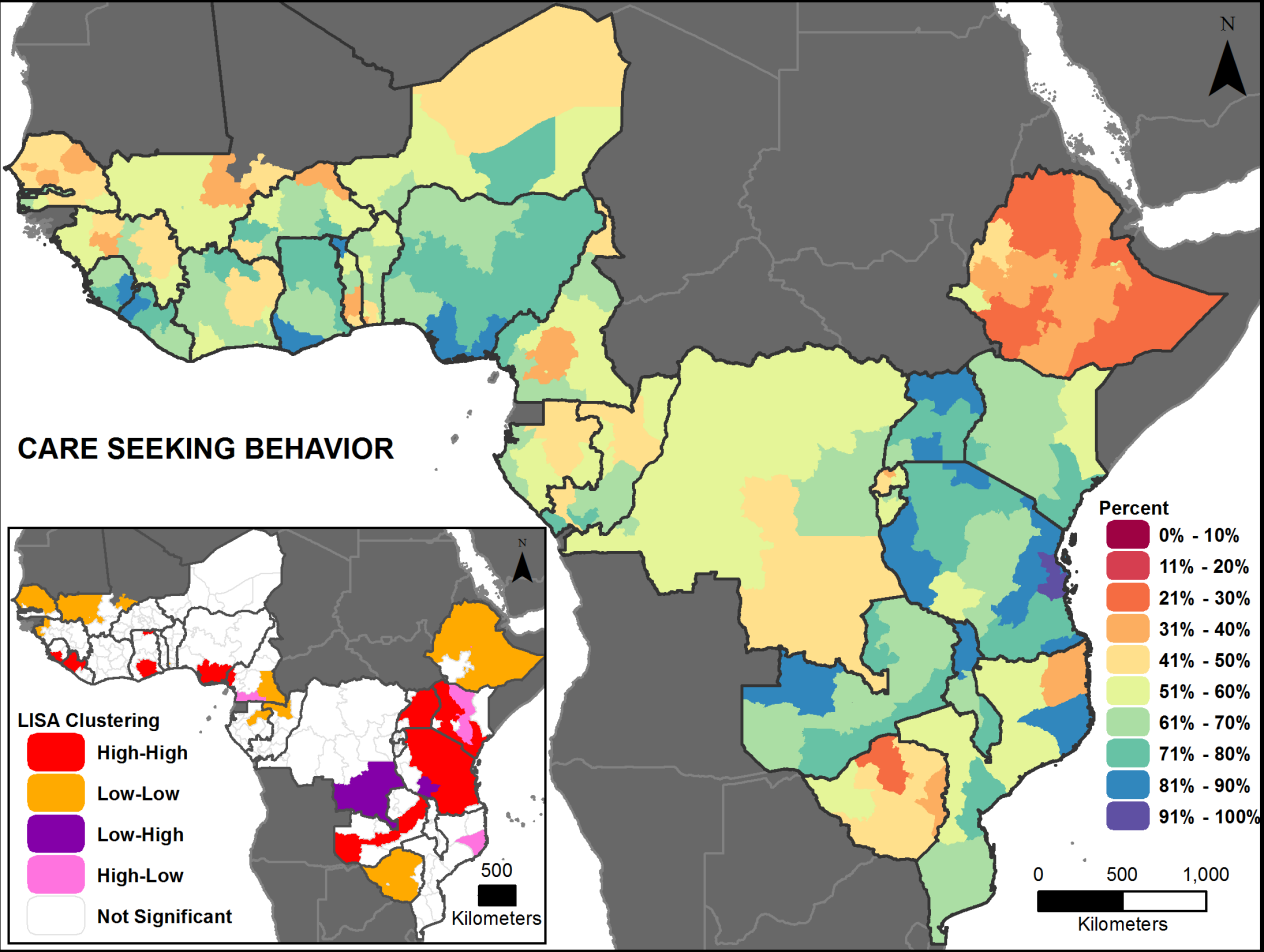
Care seeking prevalence map with LISA analysis.

**Fig 6:**
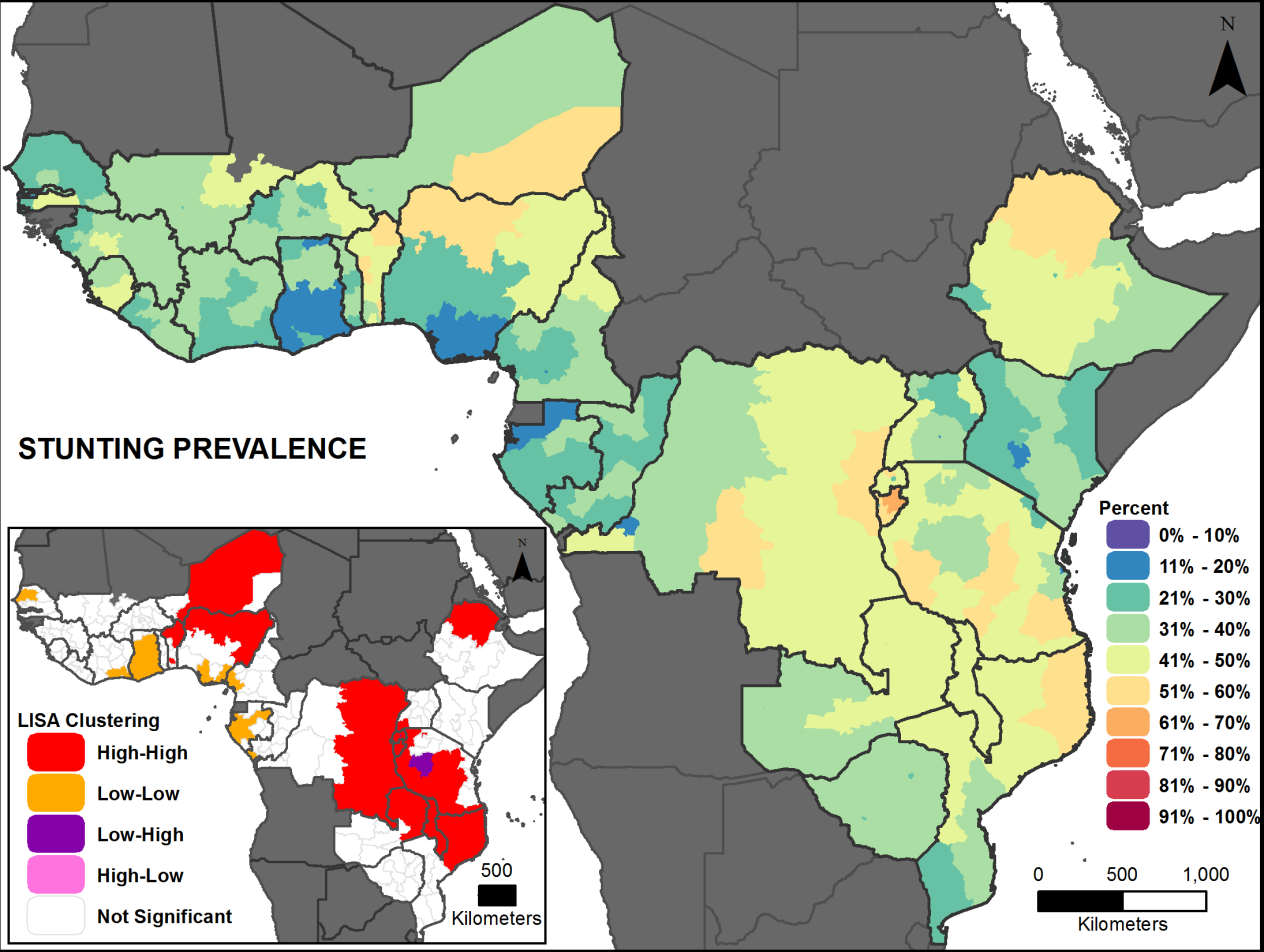
Stunting prevalence map with LISA analysis.

**Fig 7:**
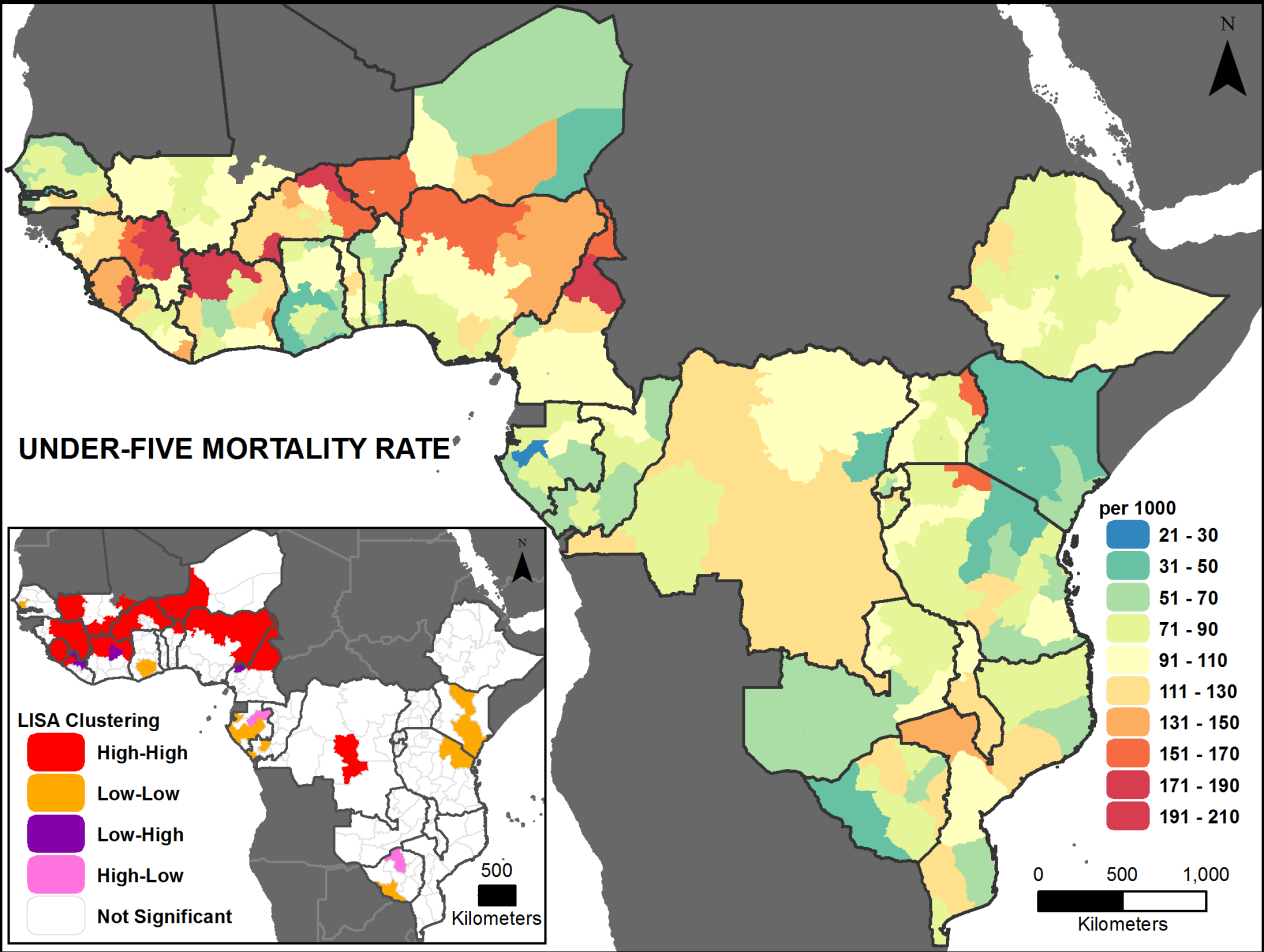
Under-5 mortality prevalence map with LISA analysis.

Figs 2, 3, 4, 5, 6 and 7 are thematic maps. Results of LISA analysis for each indicator are shown and symbolized as follows:

- High-High (red) signifies areas with spatial clustering of high indicator values (i.e., hotspots) and Low-Low (orange) signifies areas with spatial clustering of low indicator values (i.e., coldspots). These areas show statistically significant positive spatial autocorrelation, that is, regions that are surrounded by regions with similar values.
- High-Low (pink) and Low-High (purple) signify areas that are spatial outliers. These areas show statistically significant negative spatial autocorrelation, that is, regions that are surrounded by regions with dissimilar values.
- Grey areas are regions where no statistically significant spatial autocorrelation was found for an indicator.

The maps facilitate examination of the value of a region compared with the values of its neighboring regions. The hotspots and coldspots (high-high and low-low clusters respectively) tend to be grouped together in several regions, while the high-low and low-high clusters, which indicate comparative outliers, are usually a single region. This analysis highlights the relationship of indicator values between and among neighboring regions. Not all types of spatial associations exist for every indicator. The spatial clustering patterns may at times not seem to be matched to the prevalence map values (Figs 2, 3, 4, 5, 6 and 7). This is due to the categorization that is necessary in displaying continuous measures on the prevalence (thematic) map; thus, values that might be close in an absolute sense are in different categories depending on the data categorization.

### Spatial autocorrelation

The Global Moran’s *I* statistic is positive for each indicator (range: 0.41 to 0.68), and statistically significant (*p* <0.05). A statistically significant positive global Moran’s *I* suggests spatial autocorrelation across national borders, highlighting the need to examine health indicators across countries and not just within them (Table 3).

**Table 3:** Global Moran’s *I* statistics.

Indicator	I*
Exclusive breastfeeding	0.65
Measles vaccination	0.52
DPT-3 vaccination	0.68
Care-seeking behavior	0.51
Stunting	0.53
Under-5 mortality	0.41

*All *p*-values < 0.01.

Across the 27 countries, the maps (Figs 2, 3, 4, 5, 6 and 7 insets) indicate that some areas are more consistently in one of the LISA cluster categories, including some areas of the west African Sahel and eastern Africa. Across indicators, the high-high and low-low clusters often cross national borders, while the outlier areas often have at least one neighboring area in a different country. The overall standard errors for the estimates are within an acceptable range (0.0055–0.1261) for all of the indicators studied (S1 Fig). Although Low-Low and Low-High regions likely deserve increased attention, compared to High-High and High-Low regions (for all except the stunting and under-5 mortality indicators, for which the opposite is true), caution is needed when interpreting LISA results because regions that are grey (non-significant) are left out of the categorization. Those regions may actually be areas of high need. It is important to view each indicator map with its inset map to understand both the indicator prevalence and relative need in a region, compared to neighboring regions.

### Results by indicator

**Fig 8:**
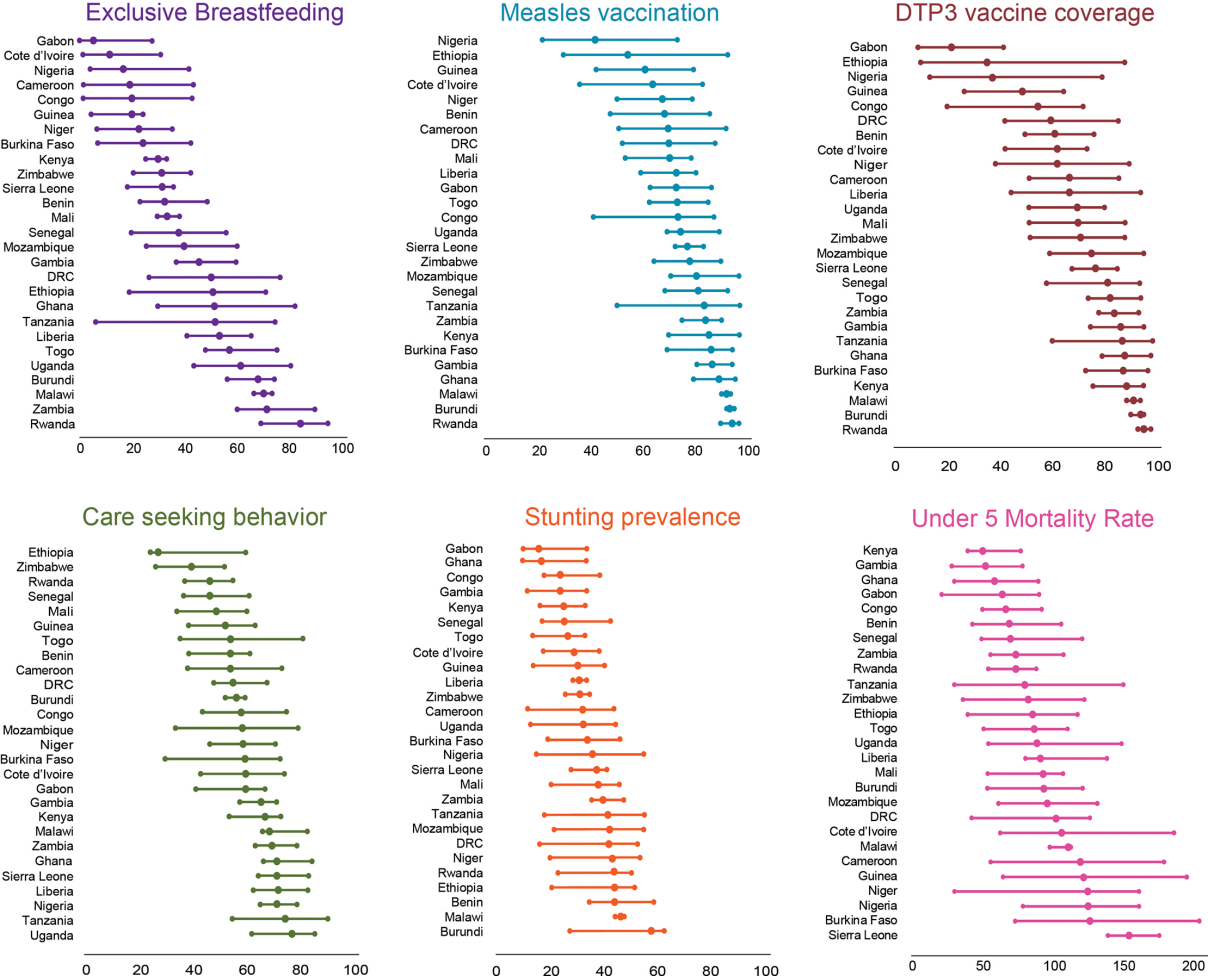
Child health indicators, plotted by lowest region, national prevalence, highest region.

There is wide variability in indicator coverage within and among countries, illustrated in Fig 8 and in the maps (Figs 2, 3, 4, 5, 6 and 7). High indicator values are “good” for four of the indicators, but “bad” for stunting prevalence and the under-five mortality rate. Ethiopia, Nigeria and Guinea were in the bottom 20 percent for four of the six child health indicators among the countries studied (Fig 8). Ghana, Malawi, Burundi, The Gambia, Rwanda, and Kenya were in the top 20% for half of the child health indicators among the countries studied (Fig 8). However, Burundi, Malawi, and Rwanda are also in the worst quintile for child stunting prevalence among the countries studied. Thus, high coverage of one or more indicators (or low stunting or mortality) does not imply high coverage of all indicators (S2 Fig).

#### Exclusive breastfeeding (EBF)

EBF prevalence ranges from 6 (Gabon) to 85 percent (Rwanda) among the countries studied, but these national estimates hide substantial subnational regional variation, e.g., from nearly 7 to 75 percent in Tanzania (Fig 8). Among the indicators studied, EBF generally had the lowest prevalence in most countries (S2 Fig). The majority of countries have intra-country prevalence differences of more than 20 percentage points. Significant positive global spatial autocorrelation exists across the study area (Moran’s *I*: 0.65, *p*-value <0.01), meaning that similar EBF values are near each other across the countries in this analysis. Fig 2 shows apparent cross-border similarities among some countries. There are high-high clusters in eastern Africa and low-low clusters in western Africa. There are a few “outlier” areas: some relatively higher areas bordering mainly low areas in western Africa, and some relatively low areas bordering higher areas in eastern Africa (Fig 2).

#### Measles vaccination

Measles vaccination coverage is generally high among the countries studied, but the range of coverage varies from an average 42 (Nigeria) to 95 percent (Rwanda), highlighting stark inequality across the study area (Fig 8). The subnational variation remains high in some countries (e.g., more than 60 percentage points in Ethiopia), but in most countries, as coverage increases at the national level, the subnational difference decreases. Burundi has the least inequality, with a coverage range from about 93 to 96 percent. Significant positive global spatial autocorrelation exists across the study area (Global Moran’s *I*: 0.52, *p*-value <0.01), meaning that similar measles vaccination coverage values are near each other across the countries in this analysis. The LISA analysis for coverage of measles vaccination identified a few areas of high-high clustering in eastern Africa and western Africa (Fig 3 inset). There are clusters of low coverage in western and central Africa, along with most of Ethiopia, and there are few apparent “outlier” areas.

#### DPT3 vaccination

Like measles vaccination, DPT3 vaccine coverage is generally high among the countries studied, but the range varies from an average of 22 (Gabon) to 97 percent (Rwanda), highlighting substantial inequality (Fig 8). Although it has the highest relative value, along with measles vaccination, among the indicators studied in each country, subnational variation remains high; most countries have a difference of more than 20 percentage points between their lowest and highest subnational area (Fig 8). Ethiopia has the widest range, from 10 to 89 percent; Rwanda has the smallest range from 94 to 99 percent. Significant positive global spatial autocorrelation exists across the study area (Global Moran’s *I*: 0.68, *p*-value<0.01), meaning that similar values are adjacent across the study area. The LISA clustering looks similar to that of measles vaccination, although the low-low areas cover a smaller space, while the high-high clusters contain more contiguous areas, with a larger stretch in eastern and western Africa (Fig 4 inset). Low-low areas are concentrated in central, western, and north-central Africa, as well as in Ethiopia, and there are few “outlier” areas.

#### Care seeking behavior

Care seeking behavior has a moderately high prevalence across the countries studied, with most countries at 50–80 percent (Fig 8). National averages vary from 29 (Ethiopia) to 78 percent (Uganda). Subnational variation is above 20 percentage points in most countries, with the highest range in Mozambique (34 to 81 percent), and the lowest in Burundi (53 to 60 percent). The global measure of spatial autocorrelation for care seeking behavior was significant (Global Moran’s *I*: 0.51, *p*-value <0.01), meaning there is a geographic pattern to care seeking behavior across the countries in this analysis. Fig 5 (inset) shows a few areas of high-high spatial association at the subnational level, with a large stretch in eastern Africa. Low-low associations appear mainly in western Africa, Ethiopia, Zimbabwe, and a small section of central Africa (Fig 5 inset). There are some high areas bordering lower areas where the high-high cluster ends, near Ethiopia. There is a low area bordering the high-high cluster in southern DRC.

#### Stunting

Stunting prevalence is high in most countries studied (> 20 percent), with some subnational regions below 20 percent. The range of stunting among the countries in the study area is from 17 (Gabon) to 58 percent (Burundi; Fig 8). Intra-country variation is high in most countries. The widest range is 16 to 55 percent (Nigeria), and the smallest range is in Malawi (45 to 48 percent; Fig 8). Significant positive global spatial autocorrelation exist across the study area (Global Moran’s *I*: 0.53, *p*-value <0.01), meaning that similar values are adjacent across the countries in this analysis. The LISA analysis shows two high-high areas in north-central and central-to-eastern Africa as well as northern Ethiopia (Fig 6 inset). Pockets of low-low clustering appear along the central and western African coastline. There is one low area bordering the high cluster in eastern Africa.

#### Under-five mortality rate

The under-5 mortality rate is moderately high across the countries studied, ranging from 52 per 1000 in Kenya to 156 per 1000 in Sierra Leone. Subnational variation is generally high, except for Malawi (99 to 113 per 1,000) (Fig 8). Burkina Faso has the widest range, from 75 to 207 per 1,000 (Fig 8). There is positive global spatial clustering seen across the study area for this indicator (Global Moran *I*: 0.41, *p*-value <0.01). Fig 7 (inset) shows high-high clustering across western Africa and in the very center of DRC. A few low-low clusters appear near the coast in parts of western and eastern Africa.

## Discussion

Ours is the first descriptive analysis of the spatial distribution of health inequalities with a set of key child health indicators both within and among half of the contiguous African countries. Highlighting spatial associations for key indicators among geographic clusters enables the identification of areas where inequalities are unnecessary and unjust, i.e., regional health inequity, within and between countries. Governments and implementing agencies can use these maps to plan service delivery and programs aimed at addressing health inequalities within a country and regionally. A few studies have looked at spatial autocorrelation for single or multiple indicators within a single country or within several countries—for example, water and sanitation indicators [55] and under 5 mortality [12]—but they did not compare different indicators among countries to produce a larger picture of child health inequalities. Reducing health inequity was not an explicit element of the MDGs but it is a major focus of the SDG-era [56] and there is a recognized need to make clear links between social determinants of health inequity, including geographic location, and observed inequalities in health interventions and outcomes [57].

Health inequities are differences in health deemed avoidable and unjust, and can be revealed through observed patterns of health outcomes across populations [57,58]. Some studies analyzed health inequity by wealth status [59,60], illustrating associations between wealth and health care coverage and outcomes. We observed stark geographic inequalities in health indicators, both within and among countries. These differences seem neither necessary nor just; thus, our analyses may point to regional health inequities.

Some indicators had large multi-country clusters of similar values, which could reflect similarities in country policies and their application; coordinated, exogenous efforts from foreign donors; cultural practices; or a combination of all of these factors. These cross-border bands may be due to cultural influences that underlie some of the indicators studied [17], and the fact that many ethnic groups reside in geographic areas that cross colonial-era geo-political boundaries that divided ethnic groups among countries [61] and shared geographic features that make access to health care, e.g., immunization campaigns, difficult.

### Exclusive breastfeeding and stunting

While there are similar adjacent values for EBF across the study area, large disparities within most countries may indicate that national policies that protect, promote, and support EBF are either not reaching everyone in need or have a differential effect on subpopulations. Stunting is affected by culturally-influenced consumption and feeding practices in addition to EBF, and we observed large areas of high rates of stunting in north-central and central-eastern Africa, with few dissimilar adjacent values across the study area. Food insecurity in Africa is exacerbated by climate change and HIV/AIDS [62], which affects geographic areas disproportionately, including effects on production and consumption patterns, with clear implications for nutritional status and health. Prolonged unfavorable weather patterns that restrict access to and availability of food for children, adolescents, and pregnant women can have lasting ill effects on health, since stunting begins in utero and has a multigenerational effect [63]. Hookworm, malaria, chronic diarrheal disease and other infections, including vaccine-preventable infections, common in tropical climates or low-income countries and impacted by geographic features can also affect nutritional status and contribute to stunting [19].

Some countries that had relatively good values for most child health indicators also had high stunting rates. Stunting reflects both mothers’ and children’s health. Living amidst conditions in which they have little autonomy, are deprived of human rights related to education, health and well-being, and are forced to marry young affects women’s health [12,64,65], and thus affects the health of children and societies generally. Where high stunting overlaps with these conditions, there is a need for systemic and societal changes to improve maternal and child health.

### Immunization

Variable immunization coverage in Ethiopia has led to recommendations to address regional variations in service delivery and access to information [66]. We observed that measles and DPT-3 immunization coverage is generally lower in more remote areas of west and central Africa, possibly indicating poor access to services, while the band of high-high coverage in eastern Africa may be related to ease of access to services and cultural acceptance. As the type of available vaccinations increases (pneumonia, rotavirus, etc.) community health workers may be commissioned to provide more vaccinations, thereby reducing access-related barriers in remote areas. Geographic analysis could pinpoint areas of low coverage to inform efficient deployment.

### Care seeking

Some indicators with clustering of high-high or low-low values across borders may be influenced more by factors other than culture, such as physical access to goods and services; geo-temporal and geo-climatic factors related to disease transmission; education and wealth [12]. For example, higher care seeking in coastal areas may be due to more geographic accessibility and local wealth from tourism and fishing. Costs related to transportation and time spent away from paid work are not as prohibitive in relatively well-off, compared to poorer, areas. The care-seeking coverage indicator may improve in subsequent surveys due to community case management programs for childhood illness in many countries in sub-Saharan Africa [67]. In these programs, community health workers diagnose and treat pneumonia, diarrhea, and malaria, and can reduce geographic barriers to care seeking. Geographical analysis could pinpoint areas of high-need to inform decisions about resource allocation and health worker deployment.

### Under-5 mortality

Lack of exclusive breastfeeding, illness caused by vaccine-preventable infections, and delayed care-seeking all contribute to under-5 mortality. In addition, environmental factors, including population density, farming systems, disease transmission patterns, proximity to urban areas, and armed conflict have been previously associated with high under-5 mortality in sub-Saharan Africa, and explained much of the country-specific variability in mortality in the western African Sahel region [12,68,69]. Root’s analysis of under-5 mortality among 20 African countries found that socio-economic variations influence the spatial pattern in western Africa, whereas greater variability in disease environments in eastern and southern Africa drove spatial patterns there [12]. The band of high under-5 mortality we observed across western Africa seems consistent with that observed by Root [12]. If his reasoning holds, then economic development has not yet been robust enough in this region to lower under-5 mortality rates. Like Root [12], we also observed lower under-five mortality along coastal areas compared to inland areas. The coastal regions are likely wealthier, and also have an observed higher prevalence of care seeking.

### Future directions to equitably improve maternal and child health

Geographic areas that have high coverage, or low stunting or mortality values, but are surrounded by outliers with low coverage, or high stunting or mortality values, require further study to understand what environmental, cultural, or political barriers negatively influence their neighbors’ health. These outlier areas may present opportunities for rapid remediation, if efforts can build upon effective strategies in the neighboring healthier areas. Qualitative research may illuminate factors that should be extended to or replicated in neighboring areas or uncover untapped resources.

Contiguous areas of low coverage or high need may indicate the presence of spatially dependent contextual factors that influence health indicators [12]. This analysis was not designed to examine the underlying reasons for differences in indicator coverage among contiguous subnational areas, nor interactions between factors, but more intensive ESDA including multilevel modeling with spatial regression techniques could further inform prioritization to improve health equity among children in sub-Saharan Africa. Future studies should also investigate the relationship between indicators, e.g., the relationship between exclusive breastfeeding and stunting, and between stunting and female autonomy. Investments in increasing the availability of geographic data through geo-referencing facility registries and providers would also facilitate geospatial analysis at the most proximal levels. Further bivariate LISA or spatial regression analyses incorporating information on environment, wealth, education, population density, or government policies, among others, would enhance understanding of the interplay between health determinants and geography. For example, a study of child mortality in Nepal identified a residual spatial pattern after controlling for individual- and community-level factors, and recommended considering efforts to reach populations where high mortality was spatially concentrated [70].

### Strengths and limitations of these analyses

A major strength of this study is that the DHS produces high quality data, which is representative at the sub-national regions used in our analyses [50], although analyzing data at the survey region level misses variation in smaller areas. As household survey data at lower geographic levels (e.g., administrative 2, district level) become more available, this type of geographical analysis could prove even more useful because, in many countries, districts form the administrative boundaries used for decentralized funding decisions. In addition, the approach of mapping indicator values using standard categories has the advantage of producing results that are easily interpretable by non-specialists but, unlike some other categorical selection techniques (e.g., jenks natural breaks), may place proximal values in different categories instead of into groups more closely resembling actual trends.

There are other limitations. This analysis could be biased by the modifiable areal unit problem, which occurs when the areas in an analysis are “artificial” in their size, i.e., if the same data were aggregated into different-sized units, the results of the analysis would be different. In addition, the varied size of subnational areas could bias the results to reflect the values of larger areas or that aggregate larger populations. The LISA results might be different if the excluded areas had recent DHS data available; some areas have only some of their neighbors included in the analysis, and it is not known if those excluded neighbors would render that area a hotspot or a coldspot. That is to say that the results for areas on the peripherals (borders) of the current analysis are likely biased, though it is difficult to say in what direction, as they have missing “neighbors” which may or may not be similar to the region in question. As more data become available, or if other data sources are combined, the geographic coverage of the analysis could be expanded. Finally, the different timing of surveys could be a limitation because the estimates for some indicators can be sensitive to specific shocks (drought, epidemics, etc.) or programmatic interventions (mass coverage campaigns, etc.). In this study all surveys were within a five-year period and all neighboring countries have indicator values that were calculated within two or three years of each other, which likely reduces the influence of shocks or interventions on indicator differences.

### Conclusions

The SDGs have an explicit focus on health inequity, signaling global recognition of the importance of considering equity in programs to improve health. WHO recognized the association of geography with inequitable intervention coverage [71] and UNICEF identified strategies to improve coverage of health interventions and reduce inequity, including increasing geographic access [72]. Evidence suggests that reducing health inequality within countries will require specific investments in identified geographic areas [73]. Geographic barriers to optimal health include distance to health services as well as climatic, social, and economic characteristics that cluster geographically, leading to a spatial distribution of health inequity. This analysis identified cross-border patterns in the values of health indicators using spatial autocorrelation techniques, which pinpoint where further research and efforts should be focused to examine and address underlying determinants of poor health indicators. Cross-border patterns of persistently poor health indicators imply a need for coordinated, multilateral efforts to address them. This analysis also identified shocking inequality within countries, indicating the need for a renewed commitment from governments and donors to prioritize addressing the health needs of underserved populations. We demonstrated the utility of geographical analysis for identifying areas of high need both within and among countries, and argued for the importance of understanding spatial distributions of health indicators in order to address health inequity. This type of analysis could be used to prioritize efforts and facilitate the efficient disposition of resources.

## Supporting information

S1 Fig Standard errors for prevalence estimates of child health indicators. These maps illustrate the range of standard errors for the prevalence estimates.
(PNG)

S2 Fig Prevalence and range estimates for child health indicators, by country. This figure presents estimates of indicator values within each country.
(PNG)
