# Supplementary figures and images for: Using geographical analysis to identify child health inequality in sub-Saharan Africa
Source: PLoS One. 2018 Aug 29;13(8):e0201870. doi: 10.1371/journal.pone.0201870 (PMC6114521; doi:10.1371/journal.pone.0201870)

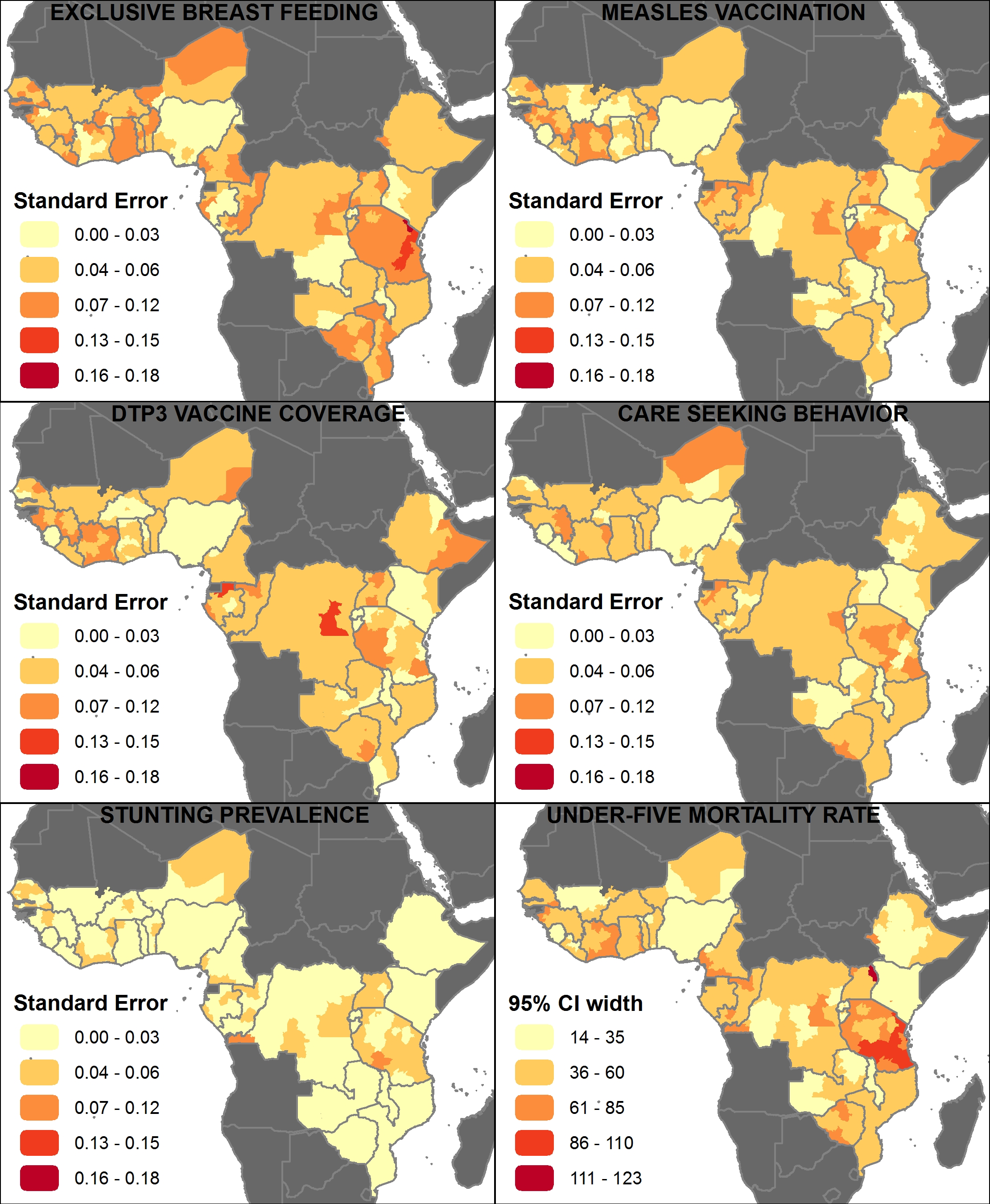

Supplement: S1 Fig — These maps illustrate the range of standard errors for the prevalence estimates. (PNG) [file pone.0201870.s001.png]

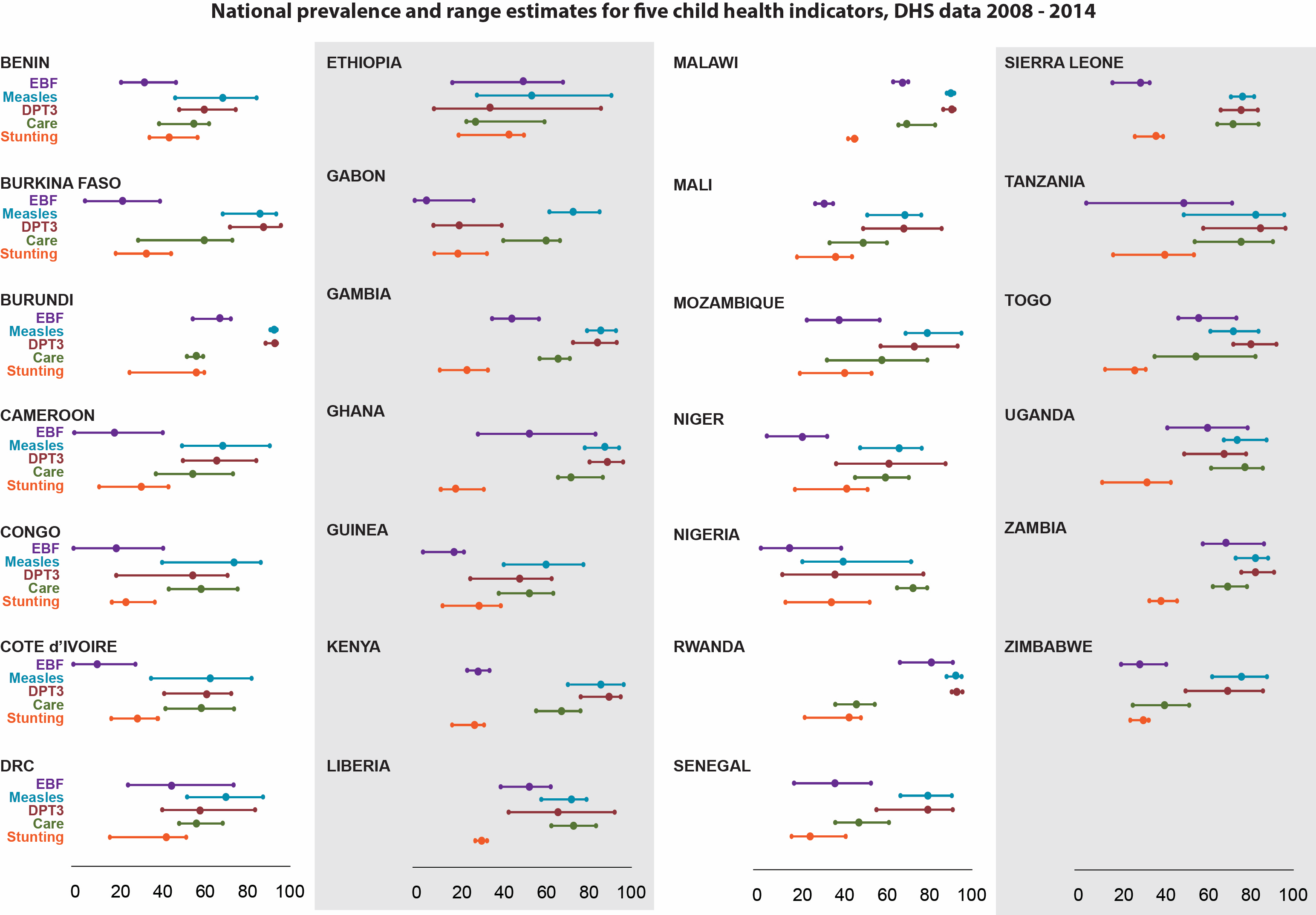

Supplement: S2 Fig — This figure presents estimates of indicator values within each country. (PNG) [file pone.0201870.s002.png]
